# The Production of Porous Asphalt Mixtures with Damping Noise Reduction and Self-Healing Properties through the Addition of Rubber Granules and Steel Wool Fibers

**DOI:** 10.3390/polym16172408

**Published:** 2024-08-24

**Authors:** Nian Chen, Huan Wang, Quantao Liu, Jose Norambuena-Contreras, Shaopeng Wu

**Affiliations:** 1 State Key Laboratory of Silicate Materials for Architectures, Wuhan University of Technology, Wuhan 430070, China; 330893@whut.edu.cn (N.C.); 303561@whut.edu.cn (H.W.);; 2 Materials and Manufacturing Research Institute, Department of Civil Engineering, Faculty of Science and Engineering, Swansea University, Swansea SA1 8EN, UK

**Keywords:** porous asphalt mixture, rubber, noise reduction, induction heating, self-healing

## Abstract

Conventional asphalt roads are noisy. Currently, there are two main types of mainstream noise-reducing pavements: pore acoustic absorption and damping noise reduction. However, a single noise reduction method has limited noise reduction capability, and porous noise-reducing pavements have a shorter service life. Therefore, this paper aimed to improve the noise-damping performance of porous asphalt mixture by adding rubber granules and extending its service life using electromagnetic induction heating self-healing technology. Porosity and permeability coefficient test, Cantabro test, immersion Marshall stability test, freeze–thaw splitting test, a low-temperature three-point bending experiment, and Hamburg wheel-tracking test were conducted to investigate the pavement performance and water permeability coefficients of the mixtures. A tire drop test and the standing-wave tube method were conducted to explore their noise reduction performance. Induction heating installation was carried out to study the heating rate and healing performance. The results indicated that the road performance of the porous asphalt mixture tends to reduce with an increasing dosage of rubber granules. The road performance is not up to the required standard when the dosage of rubber granules reaches 3%. The mixture’s performance of damping and noise tends to increase with the increase of rubber granule dosage. Asphalt mixtures with different rubber granule dosages have different noise absorption properties, and the mixture with 2% rubber granules has the best overall performance (a vibration attenuation coefficient of 7.752 and an average absorption factor of 0.457). The optimum healing temperature of the porous asphalt mixture containing rubber granules and steel wool fibers is 120 °C and the healing rate is 74.8% at a 2% rubber granule dosage. This paper provides valuable insights for improving the noise reduction performance and service life of porous asphalt pavements while meeting road performance standards.

## 1. Introduction

Cities worldwide are undergoing rapid urbanization, and the influx of people into cities has led to an increase in the density of urban populations, resulting in more and more traffic noise and a severe impact on people’s living environments [1,2]. Urbanization and increased car ownership increase traffic noise and raise the number of used tires. It is projected that by 2030, 50 billion End-of-Life tires will be discarded [3]. As people increasingly prioritize quality of life and seek more sustainable asphalt pavements, reducing traffic noise and incorporating recycled rubber tires have become key areas of research [4,5,6].

Automobile driving brings about traffic noise, and tire–road noise is one of the three main types of noise produced by vehicles [7]. The three types of noise have different proportions at different car speeds. Tire–road noise dominates at speeds above 50 km/h [8]. There are usually two ways to reduce noise: one is to do it at the source of noise generation, and the other is to intercept it through transmission. The latter requires a significant amount of capital, covers a large area, obstructs views, and affects the esthetics of the city. Therefore, addressing the issue at the source is a more scientific and effective approach. Tire–road noise can be reduced to a certain extent through design modifications.

There are three mainstream methods to reduce tire–road noise: porous sound absorption, sound wave diffuse reflection and mutual interference, and elastic noise reduction [9]. The permeable pavement disperses sound energy through its porous structure, and its surface pores and macrostructure act as air channels to release air pressure to attenuate noise generation [10]. Elastic noise reduction improves the elasticity of the pavement and attenuates the vibration between tires and the pavement, thus reducing noise [11]. While a porous structure absorbs noise, it also impairs the road performance of the material, making it challenging to improve porosity sustainably. Although elastic pavements have good damping properties, their strength is limited, and overly elastic pavements struggle to support vehicle loads. Combining these two methods provides better noise reduction.

Open-graded friction course (OGFC) has a compaction porosity of 18–25% [12]. An OGFC pavement with high porosity has good noise reduction performance [13]. Related research indicated that under different rotational speeds, OGFC reduces noise by 5.3~8.7 dB [14]. To enhance the noise reduction ability of OGFC, elastic materials are used to improve its damping effect and achieve damping noise reduction. Adding elastic rubber granules into asphalt mixtures instead of aggregates improves the overall elasticity of the asphalt mixture and enhances its driving noise reduction capability. Mixtures with rubber granules can reduce the noise generated by 2 dB [15]. Because of the high porosity of OGFC pavement, it also has good water permeability, which can quickly discharge precipitation, reduce glare, and improve the safety and comfort of driving [16]. Compared to traditional asphalt pavements, the excessive porosity of OGFC pavements and the water flow through the material make them more susceptible to water damage. Additionally, these pavements are more prone to granulation and spalling. Due to temperature variation, load, and UV exposure, pavement damage such as cracking, brittle cracking, and pitting often occurs, reducing its life span sharply [17].

Asphalt has a particular ability to heal itself. When cracks develop, the cracks will automatically heal if the asphalt pavement is given a long rest period. However, the conditions required for pavement self-healing are harsh and difficult to achieve in actual road-use environments. The flowability of asphalt can be significantly increased with induction heating, allowing it to flow into microcracks for damage repair quickly. Liu et al. demonstrated that steel wool fibers have a heating function through electromagnetic induction [18,19,20]. Electromagnetic induction heating technology can repair damage to OGFC pavement that is produced during use and increase its use time [21,22,23].

This study aims to extend the use time of OGFC pavements while improving their noise reduction effect. Firstly, the optimal oil–stone ratio of an OGFC asphalt mixture was determined and adjusted for different rubber granule dosages. Subsequently, Cantabro, immersion Marshall stability, and freeze–thaw splitting tests were conducted to characterize the particle loss and water stability performance of the mixtures. Hamburg wheel-tracking and low-temperature three-point bending tests were used to assess the high- and low-temperature properties of the mixtures. Next, a tire drop test and the standing-wave tube method were used to characterize the noise reduction performance of the mixtures. The optimum rubber granule mixing amount was decided by combining the road and the noise reduction performance test. Finally, an induction heating self-healing device was utilized to characterize the induction heating speed, optimal self-healing temperature, and maximum healing rate of the mixture.

## 2. Materials and Methods

### 2.1. Materials

The experimental coarse aggregates used in this study were basalt (5–15 mm) and limestone (0–5 mm); the fine aggregate was limestone mineral powder. The asphalt was SBS-modified asphalt. The high-viscosity modifier was produced by Jiangsu Subot New Material Co. (Nanning, China). It can be dissolved and dispersed when added to asphalt mixtures. The rubber granules were 3–5 mm in diameter and were produced from scrap tires. Steel wool fiber is used for asphalt mixture induction heating. Table 1, Table 2, Table 3 and Table 4 list the performance parameters for each material used.

### 2.2. Preparation of Asphalt Mixture

**Figure 1:**
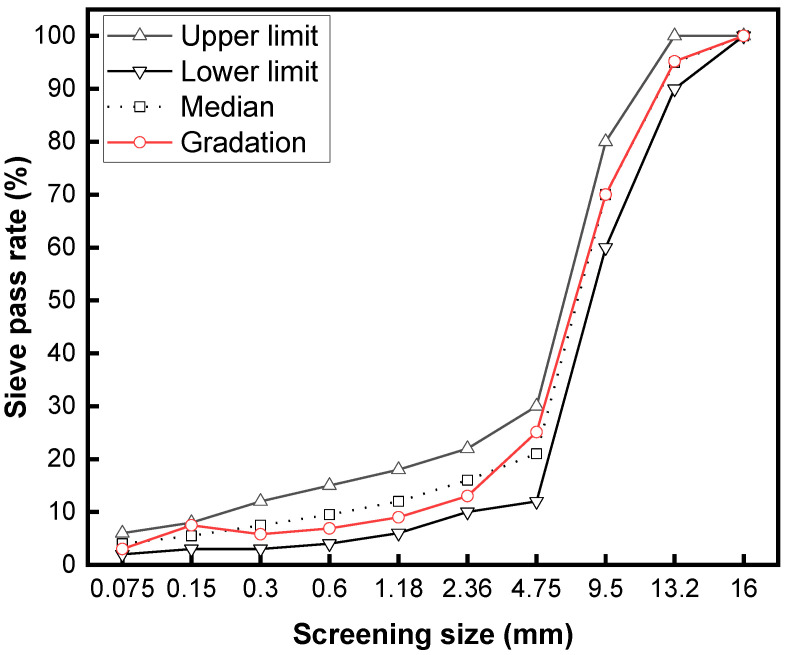
The gradation of OGFC-13.

Figure 1 demonstrates the design gradation of OGFC-13 asphalt mixtures. The optimum asphalt dosage was designed to be 4.2%, and the asphalt dosage was increased by 0.1% for each 1% increase in the addition of rubber granules. The standard Marshall specimen had a diameter of 101.6 mm and a height of 63.5 mm. Rubber granules were added as a direct equal-volume substitute for the aggregate (3–5 mm) in a dry manner. The experimental group without added rubber granules was subjected to double-sided compaction 50 times in the molding process, and the experimental group with added rubber granules was subjected to secondary compaction in the molding process. The first molding temperature was about 170 °C, and the double-sided compaction cycles were performed 40 times. The secondary molding temperature was about 80 °C, compacted 35 times on both sides [24,25]. The aggregates with rubber granules were added to the mixing pot for mixing for 90 s, and then, the steel wool fiber (6% volume of the asphalt) was added in three times with each mixing time of 30 s. The steel wool fibers were mainly used as induction heating materials to make the porous asphalt mixture induction-heatable. Excessive steel wool fibers are prone to agglomeration and tend to reduce the thickness of asphalt film, decreasing the adhesion of the asphalt and aggregate. Relevant research recommended an optimal content of steel wool fibers of 6% of the volume of asphalt [26]. A high-viscosity modifier with an 8% mass fraction of SBS-modified asphalt was added with the first steel wool fibers.

### 2.3. Porosity and Permeability Coefficient Tests

In this paper, the porosity of each mixture was measured using an asphalt mixture porosity test (volumetric method). Equations (1) and (2) were used to calculate the porosity.

(1) $$\rho_{s}=\frac{m}{v}$$

(2) $$v=\left(1-\frac{\rho_{s}}{\rho_{t}}\right)\times 100$$

where for a specimen, m (g) is the dry weight, v (cm^3^) is the volume, ρ_s_ (g/cm^3^) is the bulk density, ρ_t_ (g/cm^3^) is the maximum theoretical density of the asphalt mixtures, and vv (%) is the porosity.

The permeability coefficients of different mixtures were tested using the asphalt mixture percolation test. A pavement water penetration meter was placed on a rutted plate specimen of 300 mm × 300 mm × 50 mm molded by the wheel milling method, switched on, and logged the moments when the water surface reached 100 mL and 500 mL. The permeability coefficient was calculated according to formula (3).

(3) $$C_{w}=\frac{V_{2}-V_{1}}{t_{2}-t_{1}}$$

where C_w_ (mL/min) is the permeability coefficient, V_1_ and V_2_ (mL) represent the amount of water at each of the two timings, and t_1_ and t_2_ (s) represent the time at each of the two timings.

### 2.4. Cantabro Particle Loss and Water Stability Tests

OGFC asphalt mixture has high porosity, which leads to a more serious phenomenon of raveling and poor water stabilization properties. An asphalt mixture Cantabro test was conducted to evaluate the particle loss performance of different mixtures. An asphalt mixture Marshall stability test and an asphalt mixture freeze–thaw splitting test were used to assess the water stability.

The 24 prepared Marshall specimens were divided into six groups. One group was immersed in water at room temperature for 20 h, and another was immersed in water at 60 °C for 48 h and then left at room temperature for 24 h. The test was conducted on a Los Angeles testing machine with 300 rotations at a rotational speed of 30 r/min. The mass loss rate of each specimen was calculated using Equation (4).

(4) $$\Delta S=\frac{m_{0}-m_{1}}{m_{0}}$$

where ΔS (%) is the rate of mass loss, and m_0_ (g) and m_1_ (g) are the mass of the specimen before and after the test.

The other two sets of samples were immersed in water at 60 °C for 30 min and 48 h, and then tested for stability on a Marshall stability tester. Residual stability was calculated according to Equation (5).

(5) $$MS_{0}=\frac{MS_{1}}{MS}$$

where MS_0_ (%) is the residual stability, and MS_1_ (kN) and MS (kN) are the stabilities after 48 h and 30 min of immersion in water.

Finally, we assessed two groups of specimens, one of which was placed in an −18 °C environment for 16 h after vacuum water preservation, then placed in 60 °C water for 24 h, and finally placed in room-temperature water for 2 h together with the other group. The loading rate of the Marshall tester was set to 50 mm/min for testing the splitting strength.

The TSR was calculated according to Equations (6)–(8).

(6) $$R_{T1}=0.006278\times \frac{P_{T1}}{h_{1}}$$

(7) $$R_{T2}=0.006278\times \frac{P_{T2}}{h_{1}}$$

(8) $$TSR=\frac{R_{T2}}{R_{T1}}\times 100$$

where TSR (%) is the freeze–thaw splitting strength ratio, R_T1_ (MPa) is the splitting strength at normal temperature, R_T2_ (MPa) is the splitting strength during freeze–thaw, P_T1_ (kN) and P_T2_ (kN) are the load values of the two sets of specimens, respectively, and h_1_ (mm) and h_2_ (mm) are the heights of the two sets of specimens respectively.

### 2.5. Asphalt Mixture Bending Test

An asphalt mixture bending test was conducted to measure the low-temperature performance of different mixtures. The experimental apparatus was a universal testing machine (UTM-25). The dimensions of the specimen beam were 250 mm ± 2.0 mm long, 30 mm ± 2.0 mm wide, and 35 mm ± 2.0 mm high. The experimental temperature and loading rate were −10 °C and 50 mm/min. The maximum load was obtained by crushing the trabecular specimen. The maximum flexural–tensile stress, flexural–tensile strain, and bending stiffness modulus were calculated using Equations (9)–(11).

(9) $$R_{B}=\frac{3\times L\times P_{B}}{2\times b\times h^{2}}$$

(10) $$\varepsilon_{B}=\frac{6\times h\times d}{L^{2}}$$

(11) $$S_{B}=\frac{R_{B}}{\varepsilon_{B}}$$

where R_B_ (MPa) is the flexural–tensile strength, ε_B_ (με) is the maximum flexural–tensile strain, S_B_ (MPa) is the bending stiffness modulus at the time of specimen breakage, b (mm) is the width, h (mm) is the height, L (mm) is the span length, P_B_ (N) is the maximum load, and d (mm) is the mid-span deflection.

### 2.6. Hamburg Wheel-Tracking Test

OGFC asphalt mixtures have high porosity, so the pavement is prone to rutting, bulging, and other deformation damage. It is difficult to characterize its high-temperature performance using an ordinary rutting test, so it was characterized by the Hamburg wheel-tracking test [27,28]. The instrument produced by PWM was adopted, and the lapping wheel was made of steel. The rolling rate was 45 times/min, and the wheel load was 0.7 MPa. The experimental water bath temperature was 50 °C. When the rutting depth reached 20 mm or the number of rutting times reached 20,000, the test was terminated.

### 2.7. Damping and Noise Reduction Performance Test

Adding elastic rubber granules will give an asphalt mixture its own elastic property. An asphalt mixture with better elasticity will have a better noise reduction performance. This test evaluated the noise absorption of an asphalt mixture [29,30]. A rutting plate was placed on the ground, acceleration sensors were installed on the car tires, and the tires were dropped from a specific height to impact the rutting plate [31]. The damping noise reduction performance was determined from the acceleration decay of the tire. The better the rutting plate damping performance, the faster the acceleration decay.

The tire and rutting plate were viewed as a whole, and the following vibration equation was developed:

(12) ma+cv+kx=0

where m is the equivalent vibration mass of the tire-rutted plate system, k is the system stiffness, c is the system viscous damping coefficient, and a, v, and x are the system acceleration, velocity, and amplitude, respectively.

The solution to the equation is

(13) $$x=Ae^{-\xi t}\cos\left(\omega_{0}t+\varphi \right)$$

where A is the amplitude, ω_0_= k/m is the vibration angular frequency, ξ = c/(2m) is the vibration attenuation coefficient, t is the time, and φ is the initial phase.

The envelope equation of the solution can be presented as

(14) $$x=Ae^{-\varepsilon t}$$

It can be seen from the above equation that only the envelope equation of the vibration attenuation curve is required, and the power exponent of the equation is the vibration attenuation coefficient [32,33].

The tire used in this experiment was a 185/60R14 car tire, and its pressure was 250 kPa. The specimen was a rutting plate. The tire tread fell from 3 cm above the rutting plate, and the test equipment was a vibration experiment platform based on dSPACE.

### 2.8. Pore Noise Reduction Performance Test

This paper used the standing-wave tube method to test the vertical incidence sound absorption coefficient of different mixtures. A standing-wave tube is a cylindrical tube with a uniform cross-section, and the test specimen is the same as the inner diameter of the tube, which is the core specimen, with a diameter of 95 mm drilled from the rutting plate. When the loudspeaker emits single-frequency sound waves, these waves travel inside the tube and strike the sample’s surface. The sound waves reflect back and forth within the tube, creating a standing-wave sound field with areas of high and low intensity distributed along the axis of the tube. By measuring these high and low values [31,34], the sound absorption coefficient can be obtained using Equation (15).

(15) $$\alpha_{0}=\frac{4S}{{\left(1+S\right)}^{2}}$$

where S is the ratio of the minimal and maximal values of the sound pressure, and α_0_ (%) is the sound absorption coefficient of the vertical incidence of the sound wave.

The noise frequency of large-sized vehicles is mainly distributed under 500 Hz, the noise frequency of medium-sized vehicles is principally distributed from 500 Hz to 1000 Hz, and the noise frequency of small vehicles is principally above 1000 Hz [9,35,36,37]. Therefore, the test frequencies were categorized into the low-frequency band (400 Hz, 500 Hz), medium-frequency band (630 Hz, 800 Hz, 1000 Hz), and high-frequency band (1250 Hz, 1600 Hz). The four experimental groups were numbered 1, 2, 3, and 4 for 0%, 1%, 2%, and 3% doping with rubber granules.

### 2.9. Induction Heating Test

**Figure 2:**
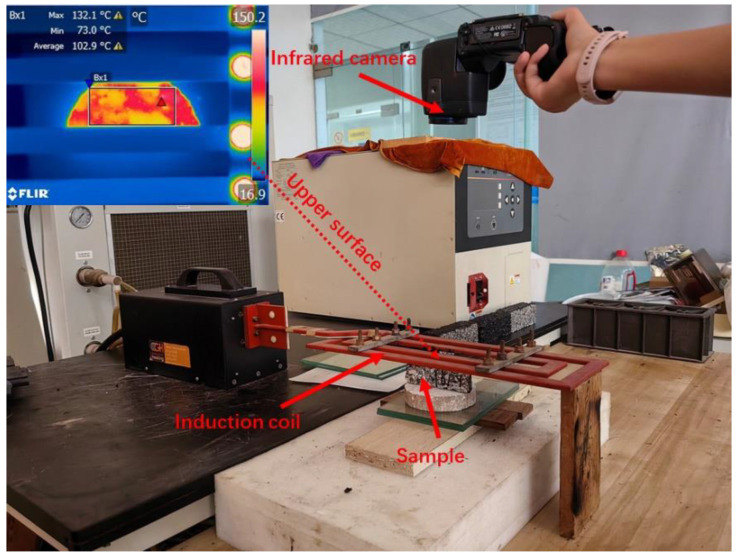
Schematic diagram of induction heating test on porous asphalt samples.

Figure 2 shows a schematic diagram of induction heating. The induction heating equipment could generate an electromagnetic field of 123 kHz. The induction coil was customized to create a 150 mm × 400 mm rectangular coil, with a coil thickness of 6 mm. A FLIR T40 infrared thermal imager was used to record the temperature of the specimen. The induction heating power was 9 kW, and the heating distance was 10 mm. Semi-circular specimens were cut from Marshall Specimens. The semicircle had a thickness of 25 mm, a height of 48 mm, and a 4 mm wide, 10 mm deep groove in the center of the bottom of the specimen. Infrared photos were taken every 10 s to record the average surface temperature of the specimen from 0 to 60 s.

### 2.10. Self-Healing Performance Test

**Figure 3:**
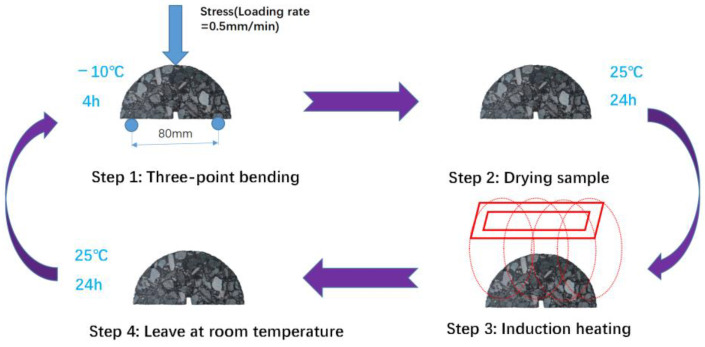
Flow chart of self-healing experiments via induction heating.

Figure 3 shows the flow chart of the self-healing experiment. A 3PB test was conducted on the specimen via a UTM to evaluate the healing level after induction heating. Firstly, the semicircular specimen was kept warm in the UTM at −10 °C for 4 h, and the 3PB test was performed on the specimen at a loading rate of 0.5 mm/min under a test temperature of −10 °C, and the peak load N_0_ was recorded. After cooling to room temperature, the specimen was heated via induction heating equipment with a heating time of 60–100 s. After cooling the specimen for 24 h, the above 3PB test was repeated and the peak load N_1_ was recorded, and the strength recovery rate was calculated from Equation (13) and used as the healing ratio of the asphalt mixture after induction heating.

(16) $$H=\frac{N_{1}}{N_{0}}\times 100\%$$

where H (%) is the strength recovery rate, N_0_ is the peak load at the first instance of damage, and N_1_ is the peak load at the second instance of damage.

## 3. Results and Discussion

### 3.1. Porosity of the Mixtures with Different Contents of Rubber Granules

**Figure 4:**
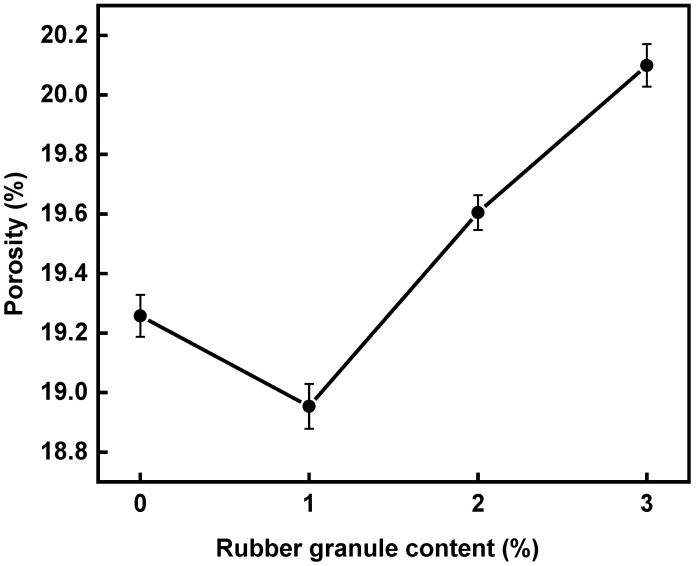
The porosity of asphalt mixtures with different contents of rubber granules.

The porosity of asphalt mixtures with four different rubber granule dosages, 0%, 1%, 2%, and 3%, is depicted in Figure 4. The porosity initially decreases and then increases with increasing rubber granule dosage, reaching a minimum value of 18.95% at a 1% dosage, meeting the porosity requirements for OGFC asphalt mixtures (>18%). This occurs because rubber granules have better compression properties than stone, resulting in compression deformation during compaction. Additionally, the elastic nature of rubber granules causes a degree of rebound after compaction. When rubber granules are added in a small amount, the rubber granules cannot easily rebound after specimen compaction. Hence, the asphalt mixture demonstrates a good compacting effect, which decreases its porosity. When too many rubber granules are added, the rubber rebound is evident, and the compacting effect is poor, which increases the asphalt mixture’s porosity.

### 3.2. Permeability Coefficients of Mixtures with Different Contents of Rubber Granules

**Figure 5:**
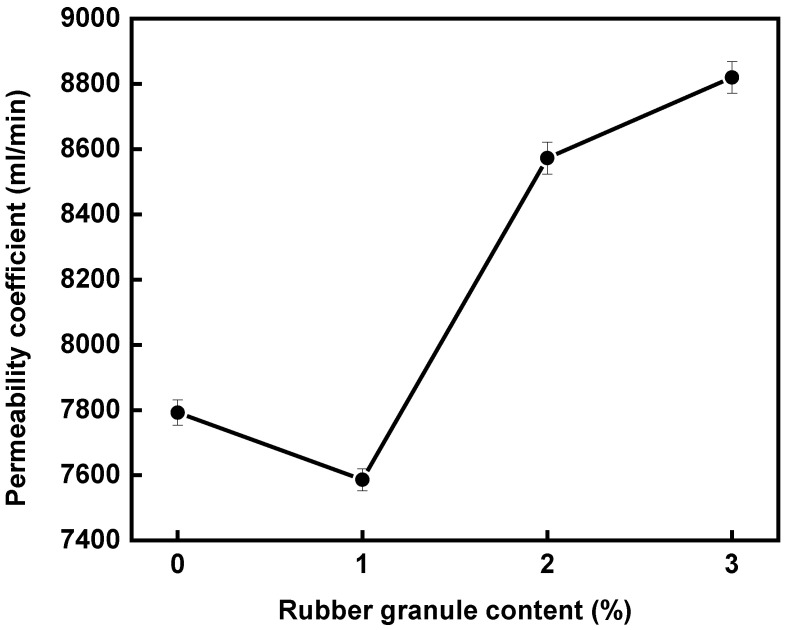
Permeability coefficients of mixtures with different contents of rubber granules.

Figure 5 illustrates the variations in the water permeability coefficient of mixtures with four different rubber granule dosages. As the rubber granule dosage increases, the water permeability coefficient first decreases and then increases, reaching a minimum value at a 1% dosage. The lowest value of the permeability coefficient is 7586 mL/min. This trend largely mirrors the trend observed in porosity. As porosity increases, the number of interconnected pores in the asphalt mixture also increases, facilitating fluid passage through the material and thereby increasing the percolation coefficient.

### 3.3. Particle Loss Rates of Mixtures with Different Contents of Rubber Granules

**Figure 6:**
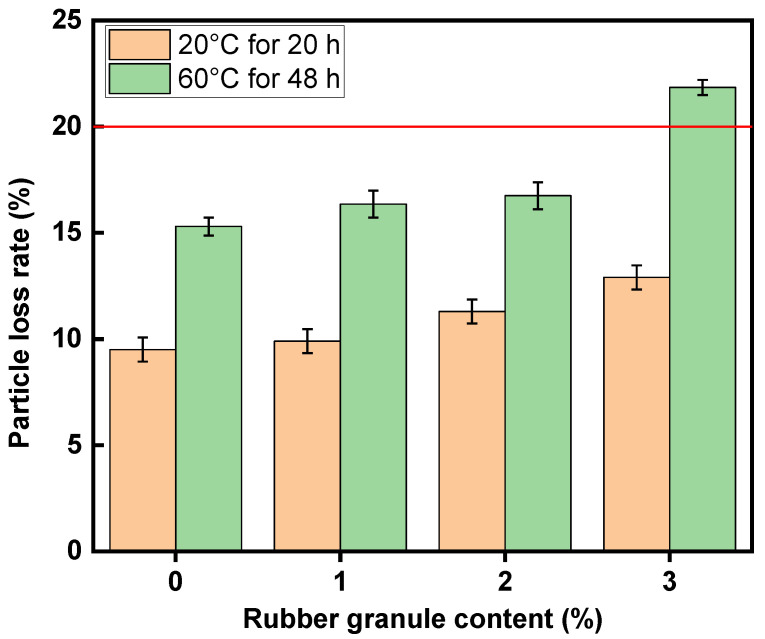
Particle loss rates of mixtures with different contents of rubber granules.

Figure 6 depicts the particle loss rates for the mixes with four different dosages of rubber. The particle loss rates of specimens after immersion at 60 °C are higher than those of specimens after immersion at 20 °C due to water damage. Moreover, as the rubber granule dosage increases, the particle loss rate of the mixture also increases. In particular, the granule loss rate of the specimen with 3% rubber granules after water immersion at 60 °C exceeds the specification requirement (20%). The rest of the specimens show particle loss rates of less than 20% and have good particle loss resistance. Hence, the rubber granule dosage should be less than 3% in this work.

### 3.4. Water Stability of the Mixtures with Different Contents of Rubber Granules

**Figure 7:**
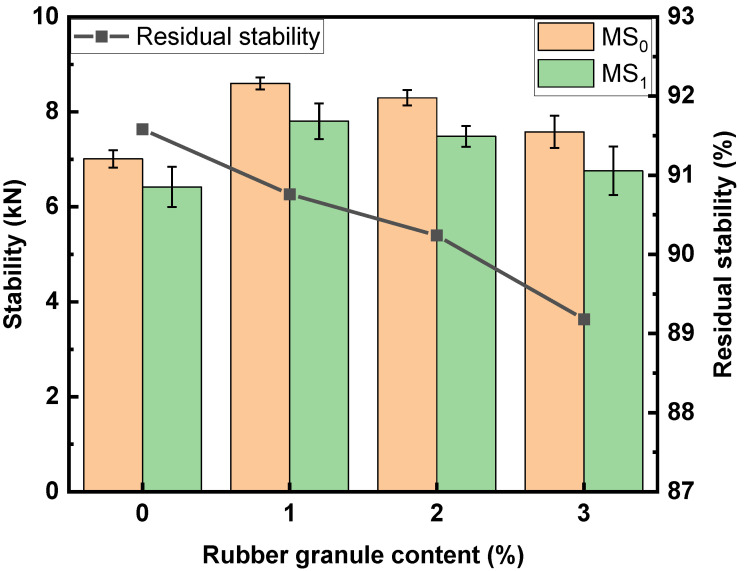
Residual stability of mixtures with different contents of rubber granules.

**Figure 8:**
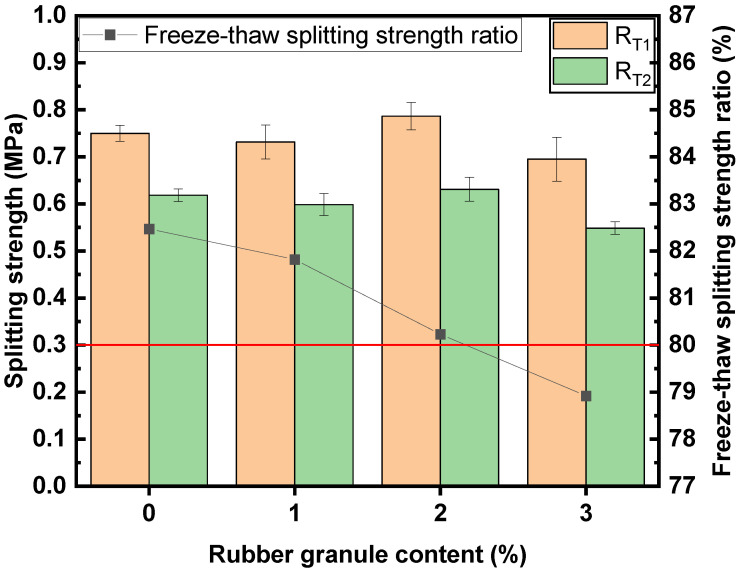
Freeze–thaw splitting strength ratios of mixtures with different contents of rubber granules.

The residual stability and TSR are depicted in Figure 7 and Figure 8, respectively. As the dosage of rubber granules increases, the residual stability and TSR decrease continuously, resulting in reduced water stability. The lowest value for residual stability is 86.2%, which is higher than the specification requirement of 85%. In particular, the TSR at a rubber granule content of 3% (78.9%) is lower than the specification requirement (80%).

### 3.5. Low-Temperature Performance of Mixtures with Different Contents of Rubber Granules

Table 5 shows the results of the 3PB experiments for the four mixes with varying rubber granule dosages. Both the flexural–tensile stress and flexural–tensile strain of the asphalt mixture decrease as the rubber granule dosage increases, while the maximum bending stiffness modulus increases. Consequently, as more rubber granules are added, the low-temperature cracking resistance diminishes. This is because porous asphalt mixtures with many coarse aggregates and fewer fine aggregates result in larger aggregate gaps and weaker aggregate bonding. Additionally, during compaction, the elastic rebound deformation of the rubber further increases the aggregate gaps, further reducing the low-temperature cracking resistance.

### 3.6. High-Temperature Performance of Mixtures with Different Contents of Rubber Granules

**Figure 9:**
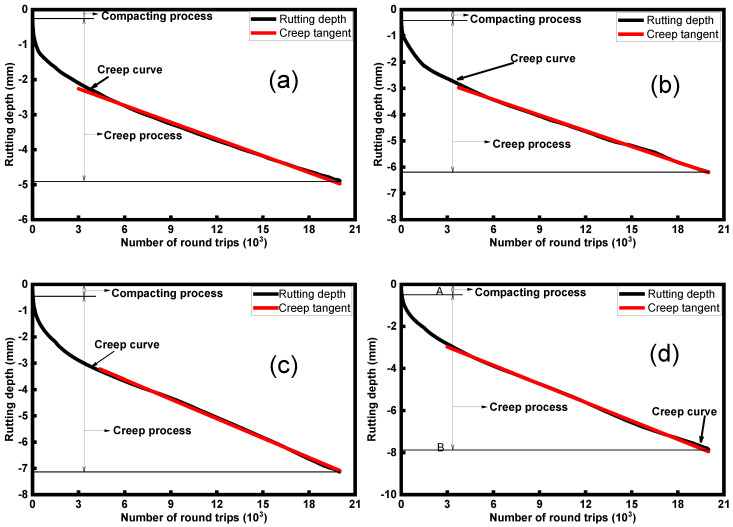
Hamburg wheel-tracking test results of different mixtures: (**a**) rubber granule dosage 0%, (**b**) rubber granule dosage 1%, (**c**) rubber granule dosage 2%, and (**d**) rubber granule dosage 3%.

The results of the Hamburg wheel-tracking test are shown in Figure 9 and Table 6. In Figure 9, the Hamburg wheel-tracking test is divided into two phases: the compaction process and the creep process. During the compaction process, the rutting depths of the four mixtures increase with higher rubber granule dosages, indicating that rubber granules impact the early compaction phase. In the creep process, the addition of rubber granules leads to a larger creep slope, which increases with the rubber granule dosage. The rising creep slope suggests that incorporating rubber granules diminishes the high-temperature rutting resistance of the mixtures. The final rutting depth increases with an increasing dosage of rubber granules and the maximum rutting depth is 7.87 mm for a 4% rubber granule dosage, which indicates that rubber granules impaired the rutting resistance of the mixtures. Moreover, the spalling process does not occur during 20,000 rounds of rutting and rolling, indicating that the asphalt mixtures have good bonding properties between the asphalt and aggregate and excellent spalling resistance.

### 3.7. Effect of Rubber Granule Content on Damping and Noise Reduction Performance of the Mixture

**Figure 10:**
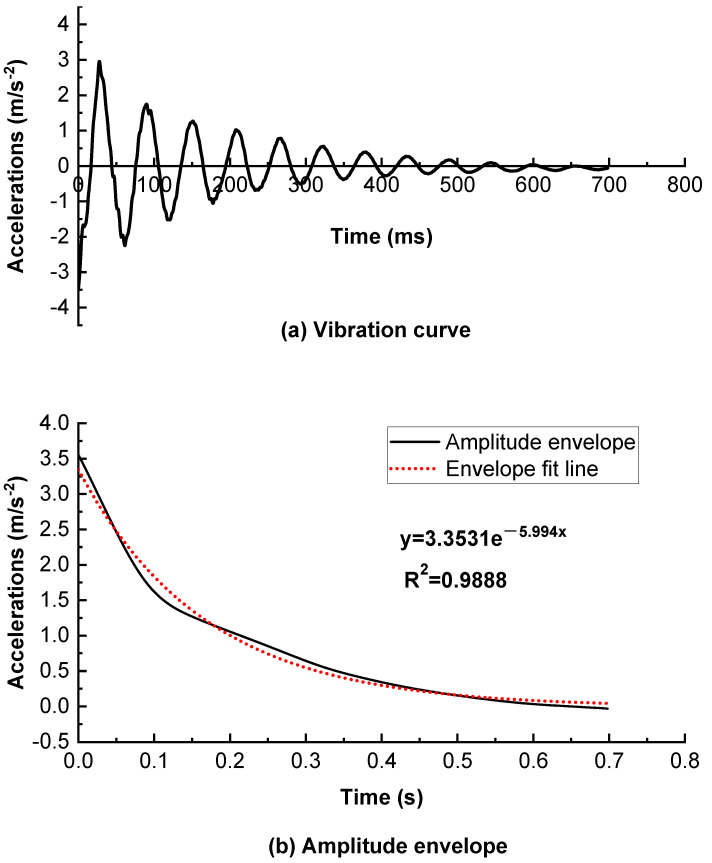
Vibration curves and amplitude envelope of tire on mixture without rubber granules.

For the four different rubber granule dosages, the first group of mixes (0% rubber granule content) was selected as an example to draw vibration curves as well as an amplitude envelope and an envelope fit line (see Figure 10) for analysis, while the rest of the groups were not explicitly analyzed. Figure 10 shows the equation of the envelope fitting line of the vibration curve. The power exponent of this equation is the vibration attenuation coefficient.

**Figure 11:**
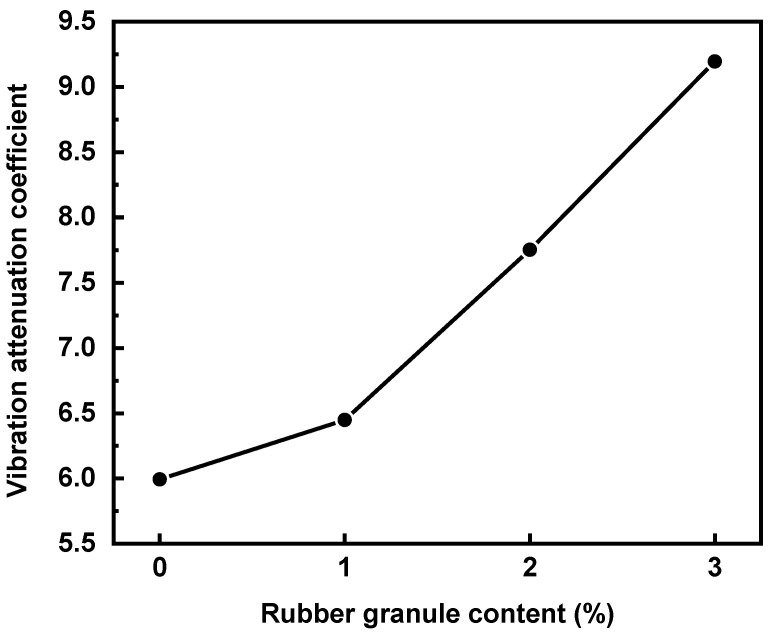
Vibration attenuation coefficients for different rubber granule dosages.

Moreover, the vibration attenuation coefficients for all experimental groups are shown in Figure 11. This figure shows that the vibration attenuation coefficient is positively correlated with the rubber granule dosage, and it increases with a rise in rubber granule dosage. It is shown that incorporating rubber granules can enhance the damping performance and the noise reduction effect. The experimental group with a 3% rubber granule dosage has the best vibration attenuation coefficient of 9.194, which is 53.4% higher than that of the 0% doping experimental group, 42.6% higher than that of the 1% doping experimental group, and 18.6% higher than that of the 2% doping experimental group.

### 3.8. Porous Noise Reduction Performance of Mixtures with Different Contents of Rubber Granules

**Figure 12:**
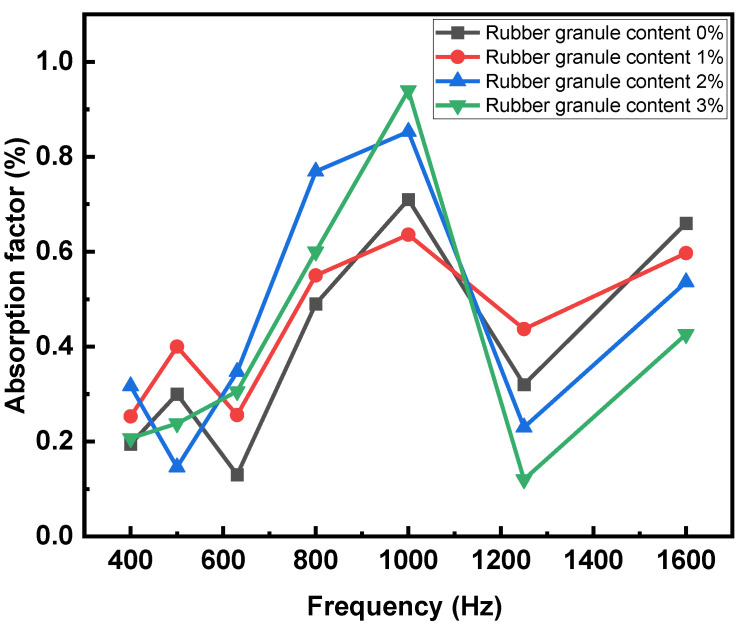
Sound absorption coefficient versus frequency for different experimental groups.

Figure 12 illustrates the data from the standing-wave tube test. In the low-frequency range, the mixture with 1% rubber granules has the best sound absorption performance, with an average absorption coefficient of 0.327, which is 31.9% higher than that of the mixture without rubber granules, 40.9% higher than that of the mixture with 2% rubber granules, and 46.6% higher than that of the mixture with 3% rubber granules. In the mid-frequency range, the mixture with 2% rubber granules has the best sound absorption performance, with an average absorption coefficient of 0.656, which is 6.7% higher than that of the mixture with 3% rubber granules, 36.4% higher than that of the mixture with 1% rubber granules, and 48.1% higher than that of the mixture without rubber granules. In the high-frequency range, the mixture with 1% rubber granules has the best sound-absorbing performance, with an average sound-absorbing coefficient factor of 0.517, which is 5.5% higher than that of the mixture without rubber granules, 35% higher than that of the mixture with 2% rubber granules, and 89.4% higher than that of the mixture with 3% rubber granules. In the peak sound absorption coefficient, the mixture with 3% rubber granules has the highest peak coefficient of 0.94, which is 10.2% higher than the mixture with 2% rubber granules, 32.4% higher than the mixture without rubber granules, and 47.8% higher than the mixture with 1% rubber granules.

The average absorption factors of different mixtures in the full frequency range are demonstrated in Table 7. The mixture with 2% rubber granules has the best sound absorption performance, with an average absorption coefficient of 0.457, which is 2.2% higher than the mixture with 1% rubber granules, 12.8% higher than the mixture with 3% rubber granules, and 14.3% higher than the mixture without rubber granules.

The sound absorption coefficient has a certain correlation with porosity, and higher porosity cause higher peak sound absorption coefficients in the experimental groups. The asphalt mixture with high porosity has better sound absorption performance in the mid-frequency band, while the mixture with low porosity has better sound absorption performance in the other two frequency bands.

### 3.9. Induction Heating Performance

**Figure 13:**
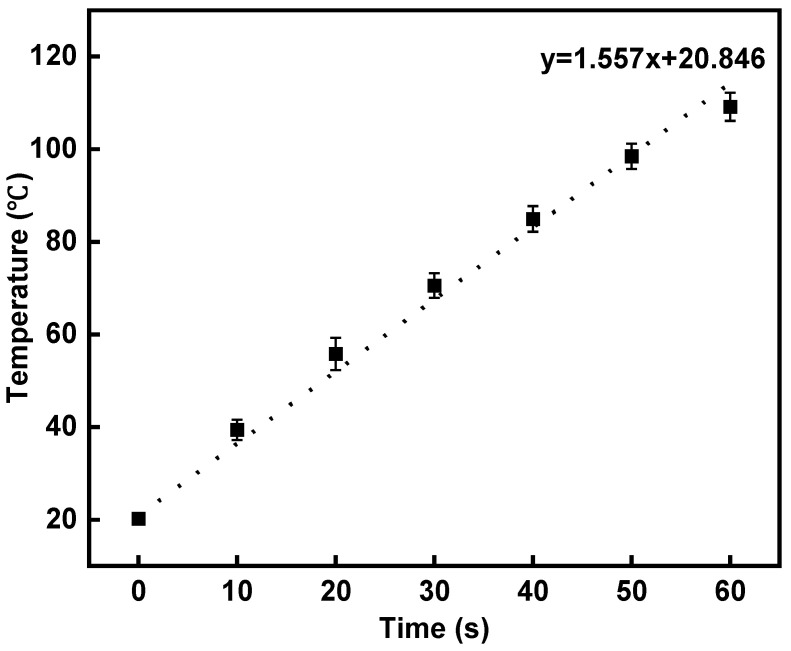
Variation in specimen surface temperature with heating time.

Combining the results from both the road performance and noise reduction performance tests, the asphalt mixture with a 2% rubber granule dosage demonstrates the best overall performance across the four experimental groups. It meets road performance requirements while also exhibiting high water permeability and effective noise reduction. Therefore, the asphalt mixture with 2% rubber granules was selected as the experimental group for the induction heating self-healing test. The induction heating performance is depicted in Figure 13. As heating time progresses, the mean surface temperature of the semicircular specimen continuously increases, reaching 109 °C at 60 s. The average heating rate is 1.56 °C/s, indicating that the noise-reducing porous asphalt mixture containing rubber granules and steel wool fibers can be rapidly heated by induction for effective crack closure.

### 3.10. Self-Healing Properties

**Figure 14:**
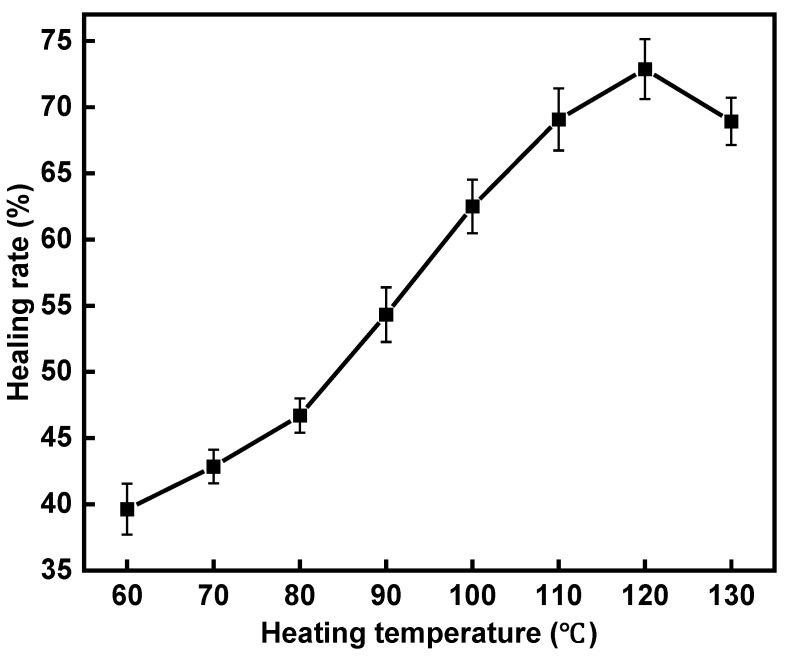
Healing rates of asphalt mixture with 2% rubber granules under different heating temperatures.

The self-healing properties are depicted in Figure 14. The healing rate of the specimens shows a tendency of increasing first and then decreasing with increasing heating temperature, with the peak occurring at 120 °C. As the heating temperature increases from 60 °C to 120 °C, the strength recovery rate increases from 39.6% to a very high value of 72.9%, indicating that the noise-reducing porous asphalt mixture with rubber granules and steel wool fibers has a good induction healing property. When the heating temperature increases to 130 °C, the healing rate decreases to 68.9%. The reason is that as the temperature of the asphalt at the crack rises, the asphalt softens and fills in the crack. When the temperature is too high, the mixture expands, causing the internal structure to break down and resulting in decreased mechanical properties, which leads to a lower healing rate.

## 4. Conclusions

This paper incorporates rubber granules into OGFC asphalt mixtures to improve noise reduction and identifies the optimal dosage of rubber granules based on road performance, drainage, and noise reduction. Additionally, induction heating self-healing technology is employed to extend the lifespan of the asphalt pavement, exploring both the induction heating and self-healing properties of the noise-reducing porous asphalt mixture containing rubber granules and steel wool fibers. The following conclusions are drawn.

(1) As the dosage of rubber granules increases, the mixture porosity initially decreases and then increases, with the lowest porosity (18.95%) observed at a 1% rubber granule content. However, as the rubber granule dosage increases, the road performance of the specimens deteriorates. At a 3% rubber granule dosage, the 60 °C immersion Cantabro particle loss and TSR exceed the specified limits for both indicators. Consequently, the water stability performance of the mixture does not meet the requirements when the rubber granule dosage reaches 3%.

(2) As the dosage of rubber granules increases, the permeability coefficient of the mixture first decreases and then increases. The porosity of the specimens with a rubber granule dosage of more than 1% increases with an increasing dosage of rubber granules, which subsequently increases the water permeability coefficient of the specimens and the drainage capacity. The higher the dosage of rubber granules, the stronger the elasticity of the specimen and the better the damping and noise reduction properties are. The porous noise reduction performance of specimens with different rubber granule dosages varies in different frequency bands, mainly focusing on 800 Hz, 1000 Hz, and 1600 Hz with higher absorption coefficients, and lower at other frequencies. The mixture with 2% rubber granules has the best overall acoustic performance.

(3) The noise-reducing porous asphalt mixture with rubber granules and steel wool fibers can be induction heated quickly. The induction heating rate of the specimen with a 2% rubber granule dosage is about 1.56 °C/s, indicating that the mixture can be induction heated quickly for self-healing.

(4) The healing rate of the noise-reducing asphalt mixture increases with the induction heating temperature, but excessively high temperatures can lead to structural breakdown of the mix and a decrease in its mechanical properties. For specimens with a 2% rubber granule dosage, the optimal healing temperature with induction heating is 120 °C, achieving a maximum healing rate of 72.9%.

This study proposed incorporating rubber granules into OGFC asphalt mixtures to combine pore noise reduction and damping noise reduction methods for enhanced noise reduction. It also investigated the use of induction heating self-healing technology to extend the lifespan of OGFC pavements. The study found that a 2% rubber granule dosage is optimal for both road performance and noise reduction. Additionally, it established the parameters for induction heating rate, optimal healing temperature, and maximum healing rate for asphalt mixtures with this rubber granule content. These findings offer valuable insights for designing such pavements and provide a basis for further research to develop noise-reducing pavements with improved performance. The parameters of the rubber granules and the induction heating process shall be optimized in future research to obtain better road performance, a more outstanding noise reduction effect, and higher self-healing efficiency.

## Figures and Tables

**Table 1:** Rubber granule performance parameters.

Technical Indicators	Apparent Density/(g/cm^3^)	Elastic Modulus/MPa	Shore Hardness/HA	Elongated Flat Granule Content/%
Test results	1.153	11.3	67	5.9

**Table 2:** Performance parameters of the SBS-modified asphalt.

Performance Indicators	Penetration at 25 °C/0.1 mm	Softening Point/°C	Ductility, 5 °C/cm	Viscosity at 135 °C/Pa·s	Densities, 15 °C/(g/cm^3^)
Test results	56	75.2	63	1.132	1.045
Requirements	40–60	≥60	≥20	≤3	-

**Table 3:** Performance index of steel wool fibers.

Technical Indicators	Diameter/μm	Length/mm	Melting Point/°C
Test results	70–130	4.2	1530

**Table 4:** Aggregate performance index.

Materials	Properties		Tested Values	Technical Requirements
Basalt	Apparent specific gravity/(g/cm^3^)	9.5–16 mm	2.980	≥2.6
		4.75–9.5 mm	2.927	≥2.6
	Los Angeles abrasion/%		10.1	≤28
	Crushed value/%		10.9	≤26
	Adhesion level		4	≥4
Limestone	Apparent specific gravity/(g/cm^3^)	3–5 mm	2.739	≥2.5
		0–3 mm	2.703	≥2.5
	Los Angeles abrasion/%		16.9	≤28
	Crushed value/%		17.2	≤26
	Adhesion level		4	≥4
Limestone Powder	Apparent specific gravity/(g/cm^3^)		2.694	≥2.5

**Table 5:** Low-temperature three-point bending test results.

Rubber Granule Content (%)	Bending and Tensile Stress (MPa)	Bending and Tensile Strain (με)	Bending and Tensile Modulus (MPa)
0	6.66	3657.50	1820.9
1	6.49	3472.70	1868.9
2	5.92	3128.89	1892.0
3	5.72	3012.65	1898.7

**Table 6:** Results of Hamburg rutting experiment.

Rubber Granule Content (%)	Deformation Depth (mm)	Creep Slope
0	4.89	−1.60 × 10^−3^
1	6.19	−1.99 × 10^−3^
2	7.10	−2.47 × 10^−3^
3	7.87	−2.93 × 10^−3^

**Table 7:** Full band absorption factor for different experimental groups.

Rubber Granule Content 0%	Full Band Absorption Factor
0%	0.401
1%	0.447
2%	0.457
3%	0.405

## Data Availability

Data will be made available on request.
